# Longitudinal early epigenomic signatures inform molecular paths of therapy response and remission in depressed patients

**DOI:** 10.3389/fnmol.2023.1223216

**Published:** 2023-08-18

**Authors:** Evelien Van Assche, Christa Hohoff, Johannes Zang, Matthew J. Knight, Bernhard T. Baune

**Affiliations:** ^1^Department of Psychiatry, University of Münster, Münster, Germany; ^2^Discipline of Psychiatry, Adelaide Medical School, University of Adelaide, Adelaide, SA, Australia; ^3^Department of Psychiatry, Melbourne Medical School, The University of Melbourne, Melbourne, VIC, Australia; ^4^The Florey Institute of Neuroscience and Mental Health, The University of Melbourne, Parkville, VIC, Australia

**Keywords:** pharmaco-epigenomics, major depressive disorder, DNA methylation, psychotherapy, pathways

## Abstract

**Introduction:**

The etiology of major depressive disorder (MDD) involves the interaction between genes and environment, including treatment. Early molecular signatures for treatment response and remission are relevant in a context of personalized medicine and stratification and reduce the time-to-decision. Therefore, we focused the analyses on patients that responded or remitted following a cognitive intervention of 8 weeks.

**Methods:**

We used data from a randomized controlled trial (RCT) with MDD patients (*N* = 112) receiving a cognitive intervention. At baseline and 8 weeks, blood for DNA methylation (Illumina Infinium MethylationEPIC 850k BeadChip) was collected, as well as MADRS. First, responders (*N* = 24; MADRS-reduction of at least 50%) were compared with non-responders (*N* = 60). Then, we performed longitudinal within-individual analyses, for response (*N* = 21) and for remission (*N* = 18; MADRS smaller or equal to 9 and higher than 9 at baseline), respectively, as well as patients with no change in MADRS over time. At 8 weeks the sample comprised 84 individuals; 73 patients had DNA methylation for both time-points. The RnBeads package (R) was used for data cleaning, quality control, and differential DNA-methylation (limma). The within-individual paired longitudinal analysis was performed using Welch’s *t*-test. Subsequently gene-ontology (GO) pathway analyses were performed.

**Results:**

No CpG was genome-wide significant CpG (*p* < 5 × 10^–8^). The most significant CpG in the differential methylation analysis comparing response versus non-response was in the *IQSEC1* gene (cg01601845; *p* = 1.53 × 10^–6^), linked to neurotransmission. The most significant GO-terms were linked to telomeres. The longitudinal response analysis returned 67 GO pathways with a *p* < 0.05. Two of the three most significant pathways were linked to sodium transport. The analysis for remission returned 46 GO terms with a *p*-value smaller than 0.05 with pathways linked to phosphatase regulation and synaptic functioning. The analysis with stable patients returned mainly GO-terms linked to basic cellular processes.

**Discussion:**

Our result suggest that DNA methylation can be suitable to capture early signs of treatment response and remission following a cognitive intervention in depression. Despite not being genome-wide significant, the CpG locations and GO-terms returned by our analysis comparing patients with and without cognitive impairment, are in line with prior knowledge on pathways and genes relevant for depression treatment and cognition. Our analysis provides new hypotheses for the understanding of how treatment for depression can act through DNA methylation and induce response and remission.

## 1. Introduction

Major depression is one of the disorders where it is well-known that disease risk is defined both by genetic vulnerability and environmental exposure (Schwabe et al., 2019). Integrating genomics and environmental information for the understanding of major depression, has become increasingly important (Ormel et al., 2019; Fabbri et al., 2020). Complementary to the genomic research, including genome-wide association studies (GWAS), Investigation of the “exposome,” with the whole of an individual’s exposure, (Vineis et al., 2020) or “phenome,” the combined set of an individual’s phenotypic features, e.g., with the emerging of phenome-wide analyses (PheWAS) are strategies that address this complexity of the genetics of the individual interacting with its environment (Sluis et al., 2010; Barch, 2022; Meng et al., 2022). How the genome and the exposome come together at the molecular level and induce changes in the individual, such as recovery from depression after a treatment, remains largely unclear. The question remains which aspects of genomics or phenomics contribute and how (Ormel et al., 2019; Matthews and Turkheimer, 2021). For genetics, focus has shifted from overall case-control design to more stratified techniques to integrate phenotypical diversity in genomic (Sluis et al., 2010; Schwabe et al., 2019). In particular for depression, stratified analyses have shown merit in advancing the field of genomics in depression at the mechanistic level of how depression treatment works. Both stratification by phenotypical characteristics of the disorder (Schwabe et al., 2019; Thalamuthu et al., 2022), but also by treatment and treatment response have helped to understand the genetic basis of depression as a psychiatric disorder (Kendall et al., 2021; Schubert et al., 2021).

The exposome has learned from genomics by adopting some of the techniques like composite scores and focusing on multiple phenotypes at once (Greener, 2019; Vineis et al., 2020; Liu et al., 2023) to understand their underlying common structure (Sluis et al., 2010; Barch, 2022; Meng et al., 2022). At some point, of course the genome and exposome act synergistically (Rappaport, 2012; Choi et al., 2022) to redefine the phenotype (Greener, 2019), e.g., a genetic vulnerability for mood disorders and stress in the environment can lead to depression. Therefore, pinpointing the mechanisms involved in this interaction is highly relevant to understand disease risk (Choi et al., 2022), but also treatment potential (Jukic et al., 2022).

One of the molecular mechanisms that is suggested to play a role in the interface between an individual’s genetic risk and the environmental exposure DNA methylation, as one example of an epigenetic mechanism (Plusquin et al., 2019; van Calker and Serchov, 2021). DNA methylation is known to be in part dependent on the underlying genomic variants, but also to be dynamic and responsive to environmental changes and alter gene expression in response to environmental influences (van Calker and Serchov, 2021; Müller et al., 2022). Therefore, it finds itself in a prime position for the investigation of early signatures of molecular change in response to treatment, such as response to a cognitive treatment. A psychotherapeutic intervention can be seen both as a treatment and an environmental factor. If successful, this environmental factor is supposed to induce early changes in the molecular functioning of the cell and the individual to induce treatment response, possibly even remission. These early molecular processes that record the environmental change and act as a switch to provide a molecular answer to the psychotherapeutic intervention and induce symptom improvement are the focus of this study. We are particularly interested in these early molecular changes in the window of the 8 first weeks of a cognitive intervention, linked to antidepressant response and remission. We want to know which loci change this early in the recovery process. These can inform an optimized treatment strategy through an early stratification of patients that may or may not respond to the chosen treatment. Therefore, we perform a case-control analysis with responders versus non-responders, but extend our analyses into within-individual longitudinal paired analyses for patients who responded or remitted to capture the dynamics of DNA methylation loci over these 8 weeks.

The opportunities of personalized medicine include person-oriented treatment choices, shortening the time to successful treatment, and early knowledge about treatment failures. We hope our results can contribute to the growing need for early indicators of treatment response. We are primarily interested in exploring the potential of early DNA methylation changes and the role DNA methylation can play in the context of treatment response, rather than understanding the precise mechanistic complexity, in the strict sense, of DNA methylation in the process of depression recovery.

Nonetheless, as depression and recovery from depression have been linked to improved neuronal connectivity (Dichter et al., 2015; Dunlop et al., 2019; Long et al., 2020), inflammation (Gasparini et al., 2022), and neurotrophic factors, such as the BDNF-pathway (Duman and Aghajanian, 2014; Li et al., 2021), we expect these signals to be represented in our pathway analysis. Due to the nature of DNA methylation and its function in gene-regulation, we also expect DNA regulatory processes to be represented in our results, as recovery from depression is a dynamic process that needs regulation at the DNA level (Mamdani et al., 2011; Woo et al., 2018).

We use the data from an existing randomized clinical trial (RCT) with depressed patients where patients received either a personalized or regular cognitive intervention. DNA methylation data are available for two time points: baseline and 8 weeks in the intervention. Therefore, this dataset is well suited to distinguish pathways that play a role in the early molecular phases of recovery: the moment where the cell is sensitive for the cognitive treatment and acts on the changed environment, hopefully for the better.

## 2. Materials and methods

### 2.1. Sample, subjects, and phenotype

Only patients diagnosed with MDD according to DSM-IV-TR were included (Knight et al., 2021). For this analysis, we used the epigenomic data of CERT-D, a previously published RCT on the effects of a cognitive intervention in patients with major depressive disorder (MDD) (Knight and Baune, 2017). Data were collected in Australia between 2017 and 2019 (Knight et al., 2021). The original sample consisted of 112 individuals. In the study all patients received an intervention to improve cognitive, emotional and functional outcomes in depression. Three individuals were excluded based on reported ancestry, which was confirmed by principal component analysis of genetic markers (Supplementary Figure 1). For this study data at baseline and the 8-week interval were used. Depression was assessed using MADRS, which was completed at baseline and after 8 weeks.

After quality control (QC), as described below, both DNA methylation data and phenotypic data were available for 90 individuals at baseline (mean age 45 years, 68% women). The mean MADRS-score at baseline was 23. At 8 weeks, the final sample with phenotypic and DNA methylation data available comprised 84 individuals (mean age 43 years, 62% women). The mean MADRS-score of the sample at 8 weeks was 15. For 73 individuals DNA methylation data at both time points were available.

For a detailed description of the trial and the sample, we refer to Knight and Baune (2017).

For this manuscript we focused on patients that showed improvement or remitted over the course of 8 weeks. In line with the available literature (Price et al., 2022), response was defined as a reduction of at least 50% of the MADRS score at baseline (*N* = 29), of which 24 individuals had DNA methylation data available at 8 weeks and 21 individuals had DNA methylation data available at both time points. Patients were considered remitted if their score after 8 weeks was smaller or equal to 9 and the baseline score was at least consistent with mild depression (MADRS score larger than 9). This was the case for 24 individuals, of which 18 individuals had DNA methylation data available at both time points. An exploratory paired longitudinal analysis was performed to provide a contrast for the longitudinal analysis independent of response. For this analysis we used all “stable” individuals who did not show a difference between baseline and 8 weeks of more than 5 points on MADRS (in either direction). A total of 28 of these individuals had DNA methylation available at both time-points and was not already included in another paired longitudinal analysis (*N* = 1). A schematic overview is presented in the Supplementary data (Supplementary Figure 2).

The study and data collection have been approved by the Human Research Ethics Committees of the Royal Adelaide Hospital and the University of Adelaide (Knight and Baune, 2017; Knight et al., 2021).

### 2.2. Epigenome-wide DNA methylation analysis

DNA methylation data (Illumina Infinium MethylationEPIC 850k BeadChip) were available of 101 individuals. Blood samples for this study were taken at baseline (T0) and after 8 weeks (T1). DNA was isolated from whole blood samples using standard procedures (QIAamp DNA Blood Midi-Kit, Qiagen, Hilden, Germany) followed by purification (Amicon 0,5ml 3K; Merck/Millipore, Darmstadt, Germany) and pipetting on 96-well plates for chip-based analyses. Bisulfite conversion and handling of the DNA methylation chips were performed in the Life & Brain Institute Bonn (Zillich et al., 2022). Samples were randomized on plates and chips based on patients’ sex and age. Both time points (T0, T1) of the same patient (for within-individual analyses) were analyzed on the same chip. Following analysis on HiScan array scanning systems (Illumina, San Diego, CA, USA), data were transferred as.idat files. The further processing and quality control of the DNA methylation data was performed using R (version 4.2.2) and the “RnBeads” pipeline [Package RnBeads 2.0, (Assenov et al., 2014; Müller et al., 2019)]. DNA methylation preprocessing was performed for baseline and 8 weeks combined, a total of 180 samples. Within-individual longitudinal analyses were performed using the Welch’s *t*-test as provided in the RnBeads package. Cross-sectional differential methylation analyses were performed with “limma,” as also embedded in the RnBeads package. To capture the dynamic aspects of early signs of treatment response in depression, we explored the overlap of the 1% most significant CpGs (i.e., 6,696 CpGs from the dataset of 669,674 CpGs) for each of the analyses as it reflects within-individual changes over the time period of 8 weeks and changes particularly linked to treatment response and remission. A detailed description of the quality control steps is provided in the Supplementary data. The same procedure was repeated for the analysis with stable individuals.

For the interpretation of results, the following online databases are used: UCSC genome browser (Lee et al., 2022), Alliance of Genome Resources (Kishore et al., 2020), and GeneCards^®^ (Safran et al., 2021).

### 2.3. Pathway analysis

The pathway analysis was performed on all 669 674 CpGs for each of the conditions. The R-package “methylGSA” (Ren and Kuan, 2019) was used for the pathway analyses as it provides statistical strategies to correct for systemic bias. The “methylglm”-command was used to extract gene-ontology (GO) terms informed by the results of the DNA methylation analyses. A minimum of 100 and maximum of 500 CpG sites per enriched term was predefined for the analyses. For the GO-analysis on hyper- or hypo-methylation [as described in the Supplementary material, Table 1 (tab 1)], the GO-analysis was repeated with either the hypo- or hyper methylated CpGs. In addition, we performed a GO analysis for longitudinal remission and longitudinal response after exclusion of the 1% (i.e., 6,696) most significant CpGs from this analysis to get a clearer image from the pathways linked to depression recovery using the stable group as a reference of longitudinal DNA methylation changes over the course of 8 weeks independent of depressive symptoms.

### 2.4. Confounding variables

Both depression and epigenome related variables were considered for the analysis. Phenotype related variables included biological sex, age, years of education as reported by the participant, body weight, and height at baseline. As ancestry was already accounted for in previous QC steps, it is not additionally represented in the final epigenome wide association analysis model. As overall missing for these variables were limited (6.08%), imputation was performed using the R package “missRanger” (v.2.1.0). Imputed variables have been additionally checked for plausibility through density plots.

Cell type deconvolution is mandatory for epigenome-wide DNA methylation analysis. As no cell type counts were available for our whole blood samples, we use a 6-cell types reference dataset as suggested by Salas et al. (2018) with the GSE110554 reference dataset using the Houseman et al. (2014) method to estimate the most important cell type fractions for our samples: neutrophils, monocytes, B-lymphocytes, natural killer cells, and CD4+ and CD8+ T-cells.

For the analysis at 8 weeks technical confounding factors (e.g., batch effects) were addressed using surrogate variables, 16 surrogate variables were estimated and included in the model, resulting in an overall genomic inflation factor (λ) = 0.97. A correlogram is also shown in the Supplementary data. For the within-individual paired analysis no confounding variables could be included in the model, as within-individual batch effects were minimized from the start, we do not expect any confounding factors to become relevant over the 8-week time period. Genomic inflation (λ) for the within-individual analyses showed a good fit for both response (λ = 0.99), remission (λ = 0.99), and the stable course (λ = 0.94).

For the exploration of baseline DNA methylation and its relation to response at 8 weeks, additional analyses were performed for the 10 CpGs as discussed in Table 2. The association between DNA methylation at baseline and MADRS at baseline was explored using linear regression analyses. These analyses too were controlled for cell-types and demographics. For response at week 8 we performed an ANCOVA with DNA methylation at baseline and response at 8 weeks, also controlled for cell-types, demographics, and MADRS at baseline.

A comparison of cell type composition for both time points is provided in the Supplementary data. Other group comparisons are shown in Table 1 and were tested using ANOVA and Fisher’s exact test.

**TABLE 1:** Descriptive statistics of the samples used for the analyses [age (years), body weight (kg), height (cm), Pers = personalized treatment, TAU = treatment as usual].

	Response 8W	Non-response 8W	Group comparison	Longitudinal response	Longitudinal remission	Stable course
*N*	24	60		21	18	28
Treatment: Pers/TAU	15/9	30/30		13/8	10/8	12/16
Age (SD)	42 (15)	44 (15)	*F*(1, 82) = 0.41, *p* = 0.52	42 (16)	46 (16)	43 (16)
Mean MADRS baseline (SD)	23 (9)	21 (9)	*F*(1, 82) = 1.75, *p* = 0.037	23 (9)	18 (8)	21 (9)
Mean MADRS difference over time	−16	−3	*F*(1, 82) = 58.91, *p* < 0.001	−16	−12	0 (3)
Mean MADRS 8W (SD)	7 (4)	18 (8)	*F*(1, 82) = 2.18, *p* = 0.0065	7.7 (4.2)	6.0 (2.7)	18 (9)
% women	16	36	*p* = 0.85	67%	67%	64%
Years of education (SD)	14 (2)	14 (3)	*F*(1, 82) = 0.26, *p* = 0.62	14 (2)	14 (2)	14 (2)
Body weight (SD)	83 (30)	76 (17)	*F*(1, 82) = 1.35, *p* = 0.25	83 (30)	83 (29)	82 (17)
Height (SD)	168 (13)	169 (8)	*F*(1, 82) = 0.13, *p* = 0.72	168 (13)	166 (11)	169 (9)

**TABLE 2:** Top 10 of response versus non-response cross-sectional analysis at week 8.

CpG	Location	% Mean methylation difference	*p*-value	Responders	Comment
cg01601845	chr3: 13296533 Open Sea	1.14	1.53 × 10^–6^	Hypo-methylated	*IQSEC1* (gene body); regulation of postsynaptic neurotransmitter receptor internalization. Located in nucleolus.
cg25153882	chr7: 117499375 Open Sea	−1.71	2.10 × 10^–6^	Hyper-methylated	*CTTNBP2* (gene body); protein coding gene. Gene-ontology (GO) annotations related to this gene include *SH3 domain binding*.
cg09148738	chr1: 153950187 Island	−0.64	4.45 × 10^–6^	Hyper-methylated	*JTB* (promotor); enables protein kinase binding activity. Involved in mitotic cytokinesis and positive regulation of protein kinase activity. Located in cytoplasm and midbody. Colocalizes with centrosome and spindle. GO annotations include protein kinase binding.
cg26340532	chr3: 32727189 South Shore	1.00	5.09 × 10^–6^	Hypo-methylated	*CNOT10*; Predicted to be involved in mRNA catabolic process and negative regulation of translation. Located in membrane. Part of CCR4-NOT complex. Among its related pathways are gene expression (transcription) and deadenylation-dependent mRNA decay.
cg01002264	chr10: 121137808 Open Sea	−1.19	1.77 × 10^–5^	Hyper-methylated	GRK5 (G protein-coupled receptor kinase 5) is a protein coding gene. Among its related pathways are GPCR downstream signaling and myometrial relaxation and contraction pathways. GO annotations include transferase activity, transferring phosphorus-containing groups and protein tyrosine kinase activity.
cg12083535	chr12: 100814114 Open Sea	−1.13	2.52 × 10^–5^	Hyper-methylated	*SLC17A8* (promotor); vesicular glutamate transporter gene. GO annotations related to this gene include symporter activity and L-glutamate transmembrane transporter activity.
cg02762115	chr11: 640446 Island	3.23	2.59 × 10^–5^	Hypo-methylated	*DRD4* (promotor); this gene encodes the D4 subtype of the dopamine receptor. GO annotations include G protein-coupled receptor activity and SH3 domain binding.
cg05625299	chr10: 1740780 North Shore	0.89	2.61 × 10^–5^	Hypo-methylated	*ADARB2* (gene-body); among its related pathways are ATP/ITP metabolism. GO annotations include RNA binding and double-stranded RNA binding.
cg01388620	chr7: 158037729 Island	−1.57	2.62 × 10^–5^	Hyper-methylated	*PTPRN2* (gene body); related pathways are innate immune system and PAK-pathway. GO annotations include phosphatase activity and transmembrane receptor protein tyrosine phosphatase activity.
cg26491461	chr1: 72748417 North Shore	1.08	2.69 × 10^–5^	Hypo-methylated	Gene desert, vicinity of *NEGR1*; predicted to act upstream of or within several processes, including positive regulation of neuron projection development.

## 3. Results

### 3.1. Sample description

Table 1 shows the descriptive statistics of the comparisons in this study. A total of 14 individuals were represented in both the longitudinal response and remission group. A schematic overview is presented in the Supplementary data (Supplementary Figure 2).

### 3.2. Response versus non-response at 8 weeks (post-intervention)

In the analysis comparing responders and non-responders with DNA methylation at 8 weeks (cross-sectional), no CpGs were genome-wide significant at the level of 5 × 10^–8^. A total of 10 CpGs had a *p*-value smaller than 3 × 10^–5^ and are shown in Table 2. The most significant CpG (cg01601845; *p* = 1.53 × 10^–6^) was located in Open Sea in the gene-body of the *IQSEC1* gene, gene linked to regulation of postsynaptic neurotransmitter receptor internalization (Kishore et al., 2020), as well as to treatment response in depression with citalopram in a prior GWAS (rs11128623) (Garriock et al., 2010). The mean methylation difference was 1.14% with relative hypomethylation for the responders.

**FIGURE 1:**
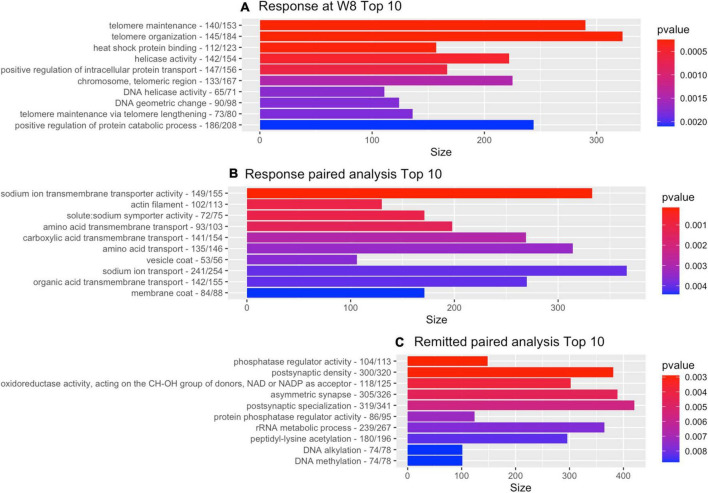
Gene-ontology pathway analysis. Top 10 of GO pathways from each of the depression-related analyses with the number of genes for the GO reference and the number of genes that contributed to the result. **(A)** Cross-sectional analysis responders vs. non-responders. **(B)** Paired within-individual longitudinal analyses with responders. **(C)** Paired within-individual longitudinal analyses with remitters.

The GO-pathway analysis returned 77 pathways with a *p*-value < 0.05. The two most significant pathways were linked to telomeres: telomere maintenance (GO: 0000723; *p* = 0.00025) and telomere organization (GO: 0032200; *p* = 0.00026) with 290 and 323 genes in the gene-set, respectively. Figure 1A shows the 10 most significant GO terms for this analysis.

A follow-up analysis on the top-10 CpGs, presented in Table 2, with DNA methylation at baseline showed no statistically significant associations with depression severity at baseline (MADRS). The analysis looking at DNA methylation at baseline and response at 8 weeks (also controlled for cell-types, demographics, and MADRS at baseline), returned 1 CpG with *p* < 0.05 (cg02762115; *p* = 0.030; *DRD4* promotor; statistical details and figure in Supplementary data, Supplementary Figure 5).

### 3.3. Within-individual paired longitudinal analysis for response

For the within-individual longitudinal analysis of responders, a total of 13 CpGs had a *p*-value smaller than 3 × 10^–5^. No CpGs were genome-wide significant at the level of 5 × 10^–8^. The most significant CpG (cg22274825; *p* = 5.10 × 10^–6^) was located at the South Shore of an island linked to the gene *SOX4*, a gene also linked to mood disorders and immune-related processes in prior research (Martinez et al., 2022). A shift was seen over time with a DNA methylation difference of 1.93%, with relative hypermethylation at 8 weeks as compared to baseline for the same individuals. The list of all 13 CpGs is presented in the Supplementary data, Supplementary Table 5.

The GO-pathway analysis returned 67 pathways with a *p*-value < 0.05. Two of the three most significant pathways were linked to sodium transport (see also Figure 1B for top 10): sodium ion transmembrane transporter activity (GO: 0015081; *p* = 0.00014; 333 genes) and solute: sodium symporter activity (GO: 0015370; *p* = 0.0011; 171 genes).

### 3.4. Within-individual paired longitudinal analysis for remission

For the within-individual longitudinal analysis of individuals that remitted over the course of 8 weeks, one CpG came close to the genome-wide significant level of 5 × 10^–8^: cg02327902 (*p* = 7.74 × 10^–8^). The CpG is located at the North Shore of an island linked to the promotor region of the *LIN37* gene, a gene with a primary role in gene regulation, cell cycle function, and mitosis (Fischer et al., 2022). A shift was seen over the course of 8 weeks with a DNA methylation difference of 1.23%, with relative hypermethylation post-intervention. Overall, 11 CpGs had a *p*-value below 3 × 10^–5^ (detailed list in Supplementary data, Supplementary Table 6).

**FIGURE 2:**
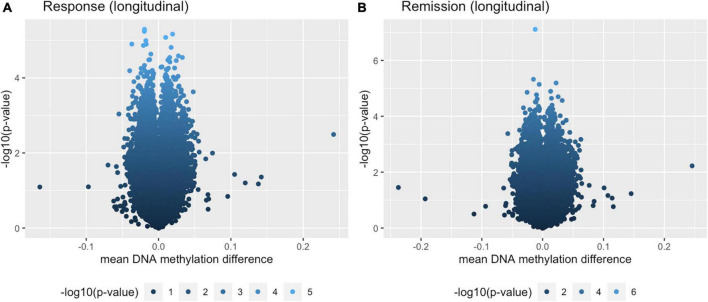
Volcano plots for both longitudinal analyses. **(A)** Within-individual paired analysis with individuals that responded over the course of 8 weeks. **(B)** Within-individual paired analysis with individuals that remitted over the course of 8 weeks.

The pathway analysis returned 46 GO terms with a *p*-value smaller than 0.05 with pathways primarily linked to phosphatase regulation (phosphatase regulator activity, GO: 0019208: 148 genes, *p* = 0.0029 and protein phosphatase regulator activity, GO: 0019888: 124 genes, *p* = 0.0072) and synaptic functioning (postsynaptic density, GO: 0014069: 381 genes, *p* = 0.0029; asymmetric synapse, GO: 0032279: 389, *p* = 0.0046; postsynaptic specialization, GO: 0099572: 420 genes, *p* = 0.0056). Figure 1C shows the 10 most significant GO terms. Figure 2 shows volcano-plots of both depression-related longitudinal analyses.

### 3.5. Within-individual paired longitudinal analysis of the stable group

The analysis for individuals not showing any change in depression severity resulted in 14 CpGs with *p* < 3 × 10^–5^. None of which overlapped with the listed CpGs of the longitudinal remission or response analysis, nor did the associated genes. From the 6,696 (top 1%) most significant CpGs from this analysis, 18 CpGs overlapped with both paired analyses and seem to contribute to a longitudinal signature. A total of 80 CpGs overlapped with the paired response analysis specifically, 93 overlapped with the paired remission analysis. These were added to the Supplementary data [Supplementary Table 1 (tab 3) and Supplementary Figure 5]. A total of 1,403 CpGs seem to be specifically linked to depression recovery processes.

At GO-analysis level, 48 GO-terms were returned with *p* < 0.05. A total of 9 GO terms of these overlapped with the paired remission analysis (*p* < 0.05). The top 10 of this GO analysis is shown in the Supplementary data [Supplementary Table 1 (tab 1)].

### 3.6. Overlapping CpGs and pathways

In the interest of finding a distinct signature of depression recovery, we looked at overlapping CpGs and pathways within the longitudinal depression-related analyses with the stable group as a contrast. As overall 669 674 CpGs were analyzed per analysis, we focused on the 6,696 most significant CpGs from each of the analyses (i.e., top 1%). The two analyses focusing on response showed 341 CpGs that overlapped. Both longitudinal analyses showed a high overlap: 1,421 CpGs. The comparison of the cross-sectional post-intervention analysis at 8 weeks and the longitudinal analysis focusing on remission returned 165 overlapping CpGs.

**FIGURE 3:**
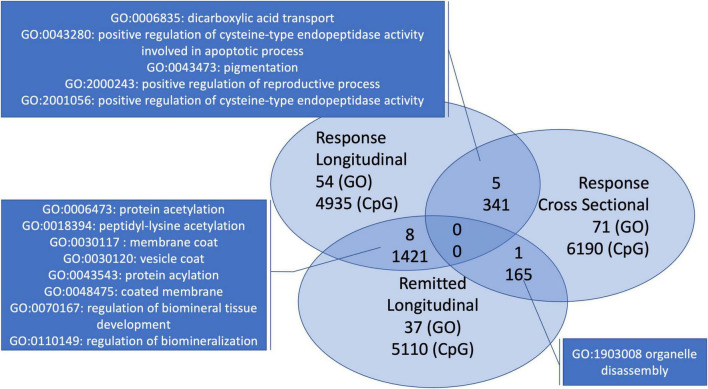
Overlapping GO-terms and CpGs for the three depression-related analyses with early treatment signatures. Both analyses looking at response have five overlapping GO-terms. The within-individual longitudinal analyses shared 8 GO-terms. The cross-sectional analysis and the longitudinal analysis with individuals that remitted have only one overlapping GO-term.

In addition, from the GO-terms analyses described in the previous paragraphs, only the ones with *p* < 0.05 per analysis were compared for overlaps. This returned 5 GO-terms overlapping between both analyses with focus on response, 8 GO-terms for the comparison of both longitudinal analyses and one overlapping GO-term for the intersection of the cross-sectional post-intervention analysis at 8 weeks and the longitudinal analysis focusing on remission (Figure 3).

Comparing with the analysis with the stable individuals, two GO-terms with *p* < 0.05 overlapped with the paired response analysis (*p* < 0.05). None overlapped within both top 10s (Figure 3), only one of *p* < 0.05 was represented in either top 10, i.e., remission (GO: 0018394 peptidyl-lysine acetylation).

Finally, we repeated the GO analysis for the paired response and paired remission analysis on all CpGs minus the top 1% CpGs (i.e., 6,676 most significant) from the so-called stable analysis. This resulted in a minor shift in priorities of GO-terms within the top-10 for each analysis with the occurrence of GO: 0098984 (neuron to neuron synapse) in the top 10 of remission and GO: 0048475 (coated membrane) in the top 10 of response [Supplementary data: Supplementary Table 1 (tab 2)].

## 4. Discussion

We performed two analyses focusing on treatment response in depression following a cognitive intervention and one analysis with focus on remission in depression. In our search for early treatment signatures in the context of treatment response and remission, we see both at the CpG and pathway (GO) level results linked to major depression recovery. The most significant gene from the analysis comparing responders and non-responders, *IQSEC1*, has been linked to treatment response to antidepressant medication at the genetic level (Garriock et al., 2010), as well as to high-risk individuals prone to developing mood disorders in a DNA methylation study (Walker et al., 2016). This result, as well as the occurrence of multiple GO-terms and pathways linked to synaptic connectivity (Dunlop et al., 2019), neurotransmitters (Saeedi et al., 2021) and DNA-related processes, such as telomeres (Hough et al., 2016) correspond to previous research and are consistent with our hypotheses. Immunological involvement was represented by the *SOX4*-gene (Martinez et al., 2022). We expected an immunological representation in the pathways and GO terms as well, which, seemed to be absent from our most important findings, however, telomerase-activity (Bürhan-Çavuşoğlu et al., 2021) and other GO terms returned by our analysis have also been linked to immunological processes, such as heat shock proteins (Srivastava, 2002). In addition, as shown in the Supplementary information (Table 2), cell-type distributions were not significantly different over the interval of 8 weeks. Therefore, the gene-regulatory and cell-internal signals related to immunological processes might be indicative of a subtle, primary reaction within these immune-cells in answer to changes in the stress system following the intervention. A follow-up analysis e.g., at 12 weeks would be useful to proof this hypothesis, maybe shows itself in a shift in the cell-type distribution within the individual. Unfortunately, we currently do not have these data available to test this hypothesis.

Also in line with our expectations is the relatively high consistency between the two analyses focusing on treatment response. The analysis comparing responders and non-responders after the intervention is different from the within-individual longitudinal analysis through the presence of a contrast group. For the longitudinal response analysis, no control group was defined as it reflects changes over time for individuals that responded. This was also the case for the analysis with remitted patients, both showed a high overlap. Only 165 of the most significant CpGs and one GO-term were shared by the remission analysis and the analysis comparing responders and non-responders. In the context of early signatures of depression treatment, where “remission” and “response” mark two stages in the continuous process, that is recovery, this might also be an indication that other genome or exposome characteristics and pathways play a role in the velocity and intensity of symptom improvement. The question can be rephrased in terms of stratification, asking who becomes either a remitter or a responder after an 8-weeks cognitive intervention. This would be in line with other literature discussing an individual’s genetic susceptibility environmental changes, either bad or good, such as a cognitive intervention, and resilience (Han et al., 2019; Marcolongo-Pereira et al., 2022). By including a reference group with individuals that did not change their depression severity over the course of 8 weeks, we could provide a “contrast” to compare the longitudinal analyses with. This contrast confirmed our results and the involvement of the specific pathways in depression recovery. The contrast with the stable individuals enforced the signals related to neuronal connectivity and cell signaling already present in the GO analyses for remission and response, through addition of neuron-to-neuron synapse, despite looking at white blood cells.

Our results did not show any genome-wide results, which can be a consequence from the limited sample sizes used for these analyses. Nonetheless, by focusing on the more homogeneous phenotypes of treatment response and remission for a longitudinal design in addition to the post-intervention cross-sectional analysis we could optimize statistical power through the paired longitudinal test. The lack of possibility to control for additional confounding variables in the longitudinal analysis is also a limitation to be addressed. However, through careful planning to avoid batch effects, the genomic inflation estimates were close to 1, which is in line with expectations of a sound analysis. Also, due to the short time-interval the variability of cell-types between both time-points, as well as other known confounders, was very limited. The course of recovery in depression is typically linked to a complex interplay of the HPA-axis, immunological processes and neurotrophic factors (Fourrier et al., 2019). We expect the DNA methylation changes to be a consequence of the intervention. Starting from that point, we assume subtle changes take place at each of these levels, leading to an overall symptom improvement in the patient, including behavioral changes.

As the dynamics of DNA methylation in a clinical context of a treatment paradigm for depression are not well understood, the investigation of early DNA methylation signatures after 8 weeks of cognitive intervention comes with the cost that the changes observed are at a preliminary stage. It is possible that we are only seeing the start of the molecular answer to the treatment and that signals will increase in significance over time. Due to these dynamic properties of DNA methylation, it is noteworthy that the results of our cognitive intervention study need replication and validation with other treatment modalities, such as medication. We also like to point out that the DNA methylation changes observed in this study, are DNA methylation changes observed in white-blood cells. Currently it is estimated, using live brain tissue after epileptic surgery, that about 20% of CpGs show a statistical correlation between the blood and the brain, saliva only 15%. Despite the higher overall correlation for saliva (*r* = 90 and *r* = 86 for blood), blood still seems to be the better, accessible proxy for CpG-specific DNA methylation (Braun et al., 2019). As stated in the introduction, we are more interested in the biomarker potential, using our small sample size. We see our results primarily as indicators of DNA methylation dynamics in an accessible tissue, such as blood, which at some point may contribute to the development of early markers for therapy response. However, our results are in line with the vast existing literature on therapy response, including research focusing on genomics (Garriock et al., 2010) and medication treatment (Garriock et al., 2010).

In the overarching context of personalized medicine, early therapy signatures that point at a mechanistic understanding of early treatment response are of particular relevance. DNA methylation and early signatures of treatment response at the molecular level can help stratify individuals by expected treatment outcome and, if necessary, support the decision for a timely treatment adaptation. Research like ours helps to finetune the interpretation of early DNA methylation changes in response to treatment. In analogy to pharmaco-epigenomics (Banerjee, 2022), also non-pharmacological treatment can induce DNA methylation changes. This knowledge is relevant topics for future research in this field, as it increases the ability to distinguish between DNA methylation responses per treatment modality for depression (Hack et al., 2019). A better understanding of early DNA methylation signatures of treatment response, or lack of molecular answer to treatment, can inform and improve patient care through a shorter time-to-decision interval. The ability to adapt treatment choices to the individual and optimize timing based on early DNA methylation signatures, can increase patient wellbeing significantly as it provides an opportunity for the acceleration of an individually optimized treatment-timeline.

## Data availability statement

The datasets presented in this article are not readily available to protect participant privacy. This is in line with the study protocol and consent procedures, as approved by the Human research ethics committees of the Royal Adelaide Hospital and the University of Adelaide. Requests to access the datasets should be directed to Bernhard.Baune@ukmuenster.de.

## Ethics statement

The studies involving human participants were reviewed and approved by the Human Research Ethics Committees of the Royal Adelaide Hospital and the University of Adelaide. The patients/participants provided their written informed consent to participate in this study.

## Author contributions

BB, MK, and EV contributed to conception and design of the study and analyses. CH and JZ organized the database. EV performed the statistical analysis and wrote the first draft of the manuscript. BB, CH, MK, and JZ wrote sections of the manuscript. All authors contributed to manuscript revision, read, and approved the submitted version.
