# Supplementary material for: Longitudinal early epigenomic signatures inform molecular paths of therapy response and remission in depressed patients
Source: Front Mol Neurosci. 2023 Aug 18;16:1223216. doi: 10.3389/fnmol.2023.1223216 (PMC10472456; doi:10.3389/fnmol.2023.1223216)
Supplement: Supplementary file 1 [file Data_Sheet_1.docx]

Supplementary Material

**Longitudinal early epigenomic signatures inform molecular paths of therapy response and remission in depressed patients**

Evelien Van Assche, Christa Hohoff, Johannes Zang, Matthew J. Knight, Bernhard T. Baune*

*** Correspondence:** Corresponding Author: [Bernhard.baune@ukmuenster.de](mailto:Bernhard.baune@ukmuenster.de)

**Sample**

**Baseline:**

Overall sample at baseline: *N*=112

Phenotype data: *N*=102

DNA methylation available (Illumina EPIC-idat): *N*=101

Following QC, five additional samples were removed: *N*=96

- Technical issues and issues and bisulfite conversion controls: 3 samples
- Ethnicity outliers: 2 samples (fig. S1)

**8 Weeks interval (8W)**

DNA methylation (Illumina EPIC) and phenotype available: *N*=89

Following QC, five additional samples were removed:

- Technical issues and issues and bisulfite conversion controls: *N*=3
- Ethnicity outliers: *N*=2

Total sample available (DNA methylation and Phenotype data): *N*=84


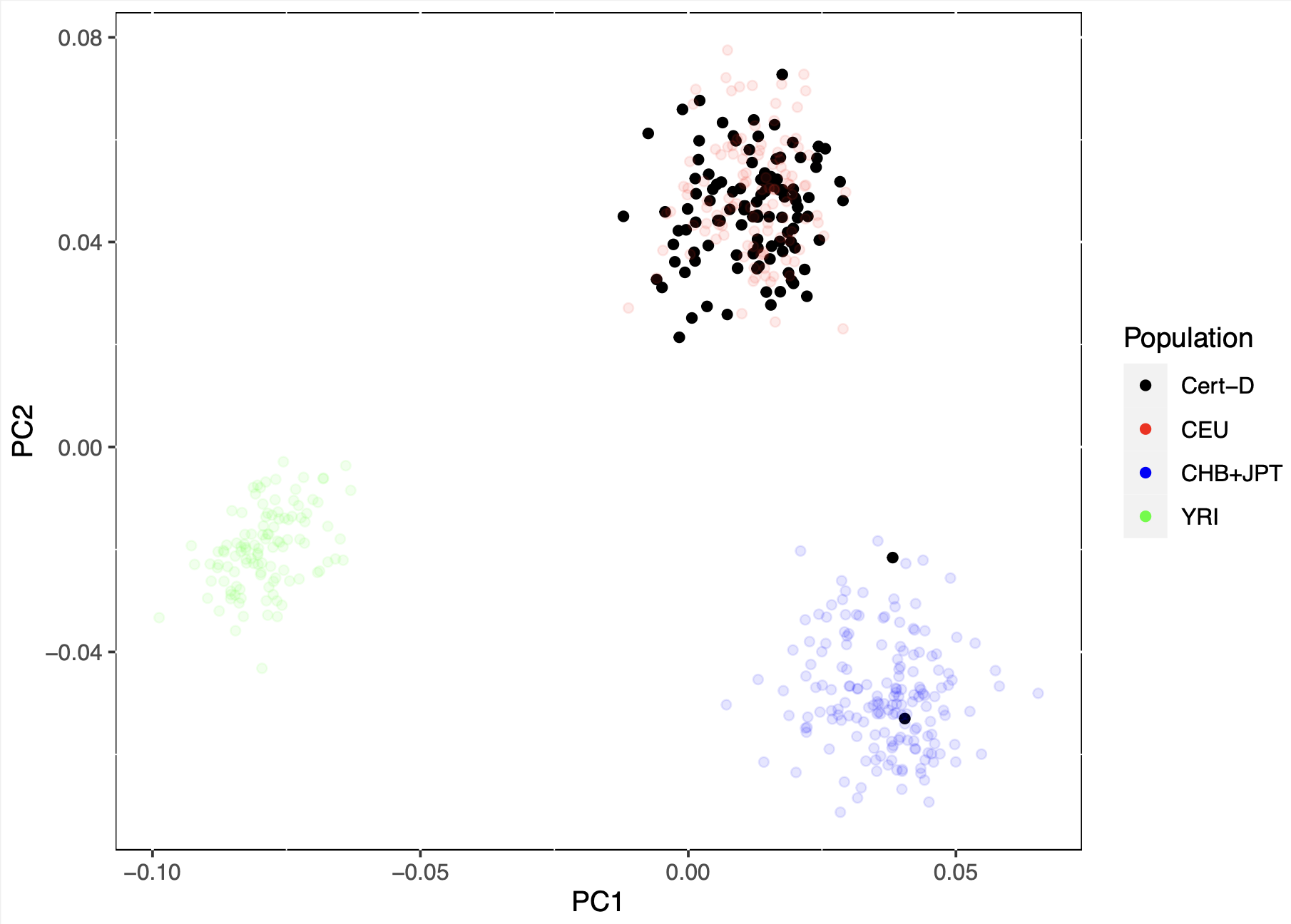


Figure S1 Ancestry comparison

Ancestry was estimated using available SNP data. Data-preparation and principal component composition were performed using PLINK^1^ and R according to the protocol by Anderson et al.^2^.


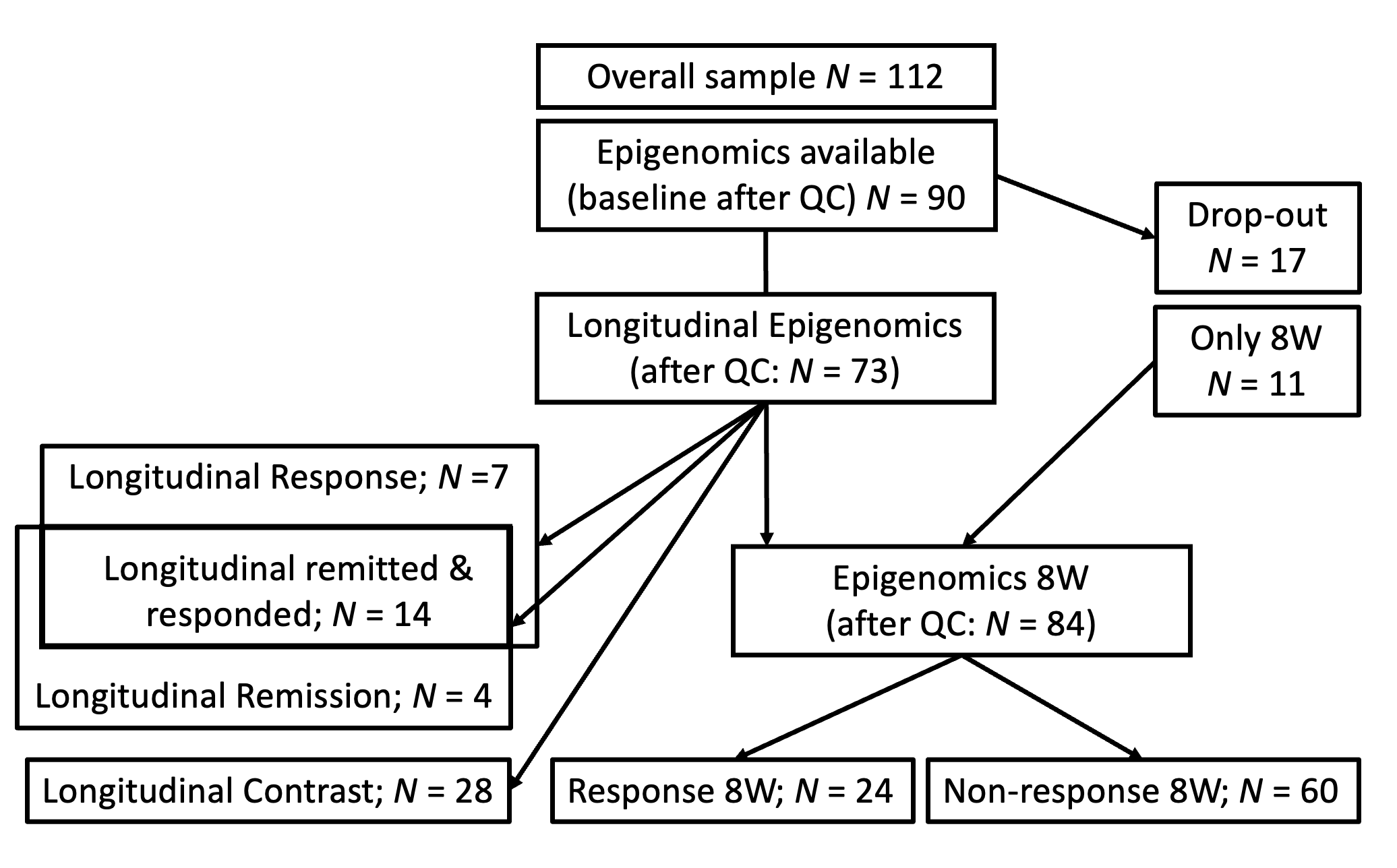


**Figure S2: Description of the samples for the different analyses**

**QC (Baseline & 8W)**

QC: bisulfite conversion and ancestry: 5 samples exluded for T1, 5 samples excluded for T2

Settings for preprocessing:

Normalisation method: dasen normalisation (WateRmelon)

Greedycut: detection p-value cut-off 0.05

Removal of: SNPrelated CGs, Sexrelated CGs, crossreactive probes

**Supplementary Table S1: QC Procedure**

|  | **Probes at start** | **Samples at start** |
| --- | --- | --- |
| **Total at start preprocessing** | **866.895** | **180** |

| **Step (Filtering I)** | **Probes removed** | **Samples removed** |
| --- | --- | --- |
| Removal of SNP-enriched Probes | *139.721* | *0* |
| Removal of Cross-reactive Probes | *34.264* | *0* |
| Greedycut | *5.643* | *0* |
|  |  |  |
| *Total removed* | *179.628* | *0* |

|  | **Probes retained** | **Samples retained** |
| --- | --- | --- |
| **Total retained** | **687.267** | **180** |

| **Step (Filtering II)** | **Probes removed** | **Samples removed** |
| --- | --- | --- |
| Context-specific Probes | *1.101* | *0* |
| Removal of Probes on Sex Chromosomes | *16.336* | *0* |
| Probes with Missing values > 90% | *156* | *0* |
|  |  |  |
| *Total removed* | *17.593* | *0* |

|  | **Probes retained** | **Samples retained** |
| --- | --- | --- |
| **Total retained** | **669.674** | **180** |

**Cell type deconvolution and handling of confounding variables**

Method: both the target dataset (CERT-D) and the dataset with the DNA methylation reference for the estimate of cell-type fractions were analysed from IDAT-files and parallel, but uniformly prepared and preprocessed with the same settings.

The IDAT-files from the 6 celltypes as discussed by Salas et al. ^3^, reference dataset GSE110554. For our estimates we discarded the artificial mixes and only used the six isolated cell-types for a total of 37 samples: Neutrophils (N=6), Monocytes (N=6), B lymphocytes (N=6), CD4+ T-cells (N=7), CD8+ T-cells (N=6), and natural killer cells (N=6)

Both datasets were combined for the estimate of cell type fractions using the RnBeads built-in function ‘rnb.execute.ct.estimation()’.

Celltype estimates were added to the phenotype and confounding variables. Based on the pre-defined outcome variables, RNBeads suggested 16 surrogate variables for cross-sectional analysis at 8 weeks. These were included in the model. The hidden variables mainly captured variation from a technical origin (Sentrix), as shown in the correlogram.

**Supplementary Table S2: Comparison of cell-type distributions over time (8 weeks)**

ANOVA per Cell-type comparing both Time-points.

|  | Paired Response | Paired Remission |
| --- | --- | --- |
| Neu | *F*(1,40)=0.01; *p*=0.93 | *F*(1,34)=0.37; *p*=0.55 |
| Mono | *F*(1,40)=1.09; *p*=0.30 | *F*(1,34)=0.27; *p*=0.61 |
| NK | *F*(1,40)=0.84; *p*=0.37 | *F*(1,34)=1.82; *p*=0.19 |
| Bcells | *F*(1,40)=0.35; *p*=0.56 | *F*(1,34)=0.21; *p*=0.65 |
| CD4T | *F*(1,40)=0.63; *p*=0.43 | *F*(1,34)=1.07; *p*=0.31 |
| CD8T | *F*(1,40)=2.10; *p*=0.16 | *F*(1,34)=0.94; *p*=0.34 |

**Supplementary Figure S3: QQ-plots**

A): cross-sectional analysis at 8 weeks (response vs. no response)
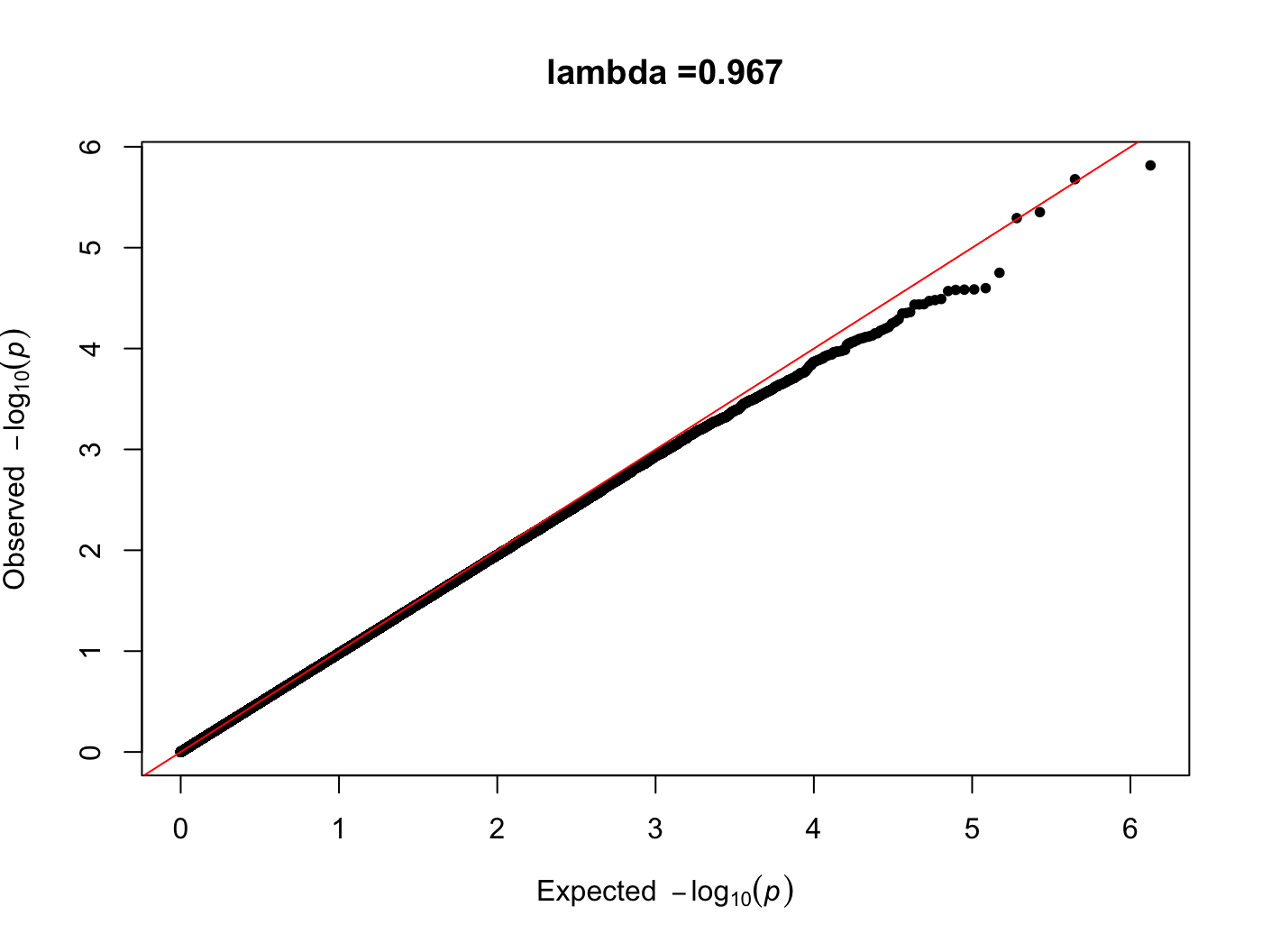


B): paired within-individual analysis for response
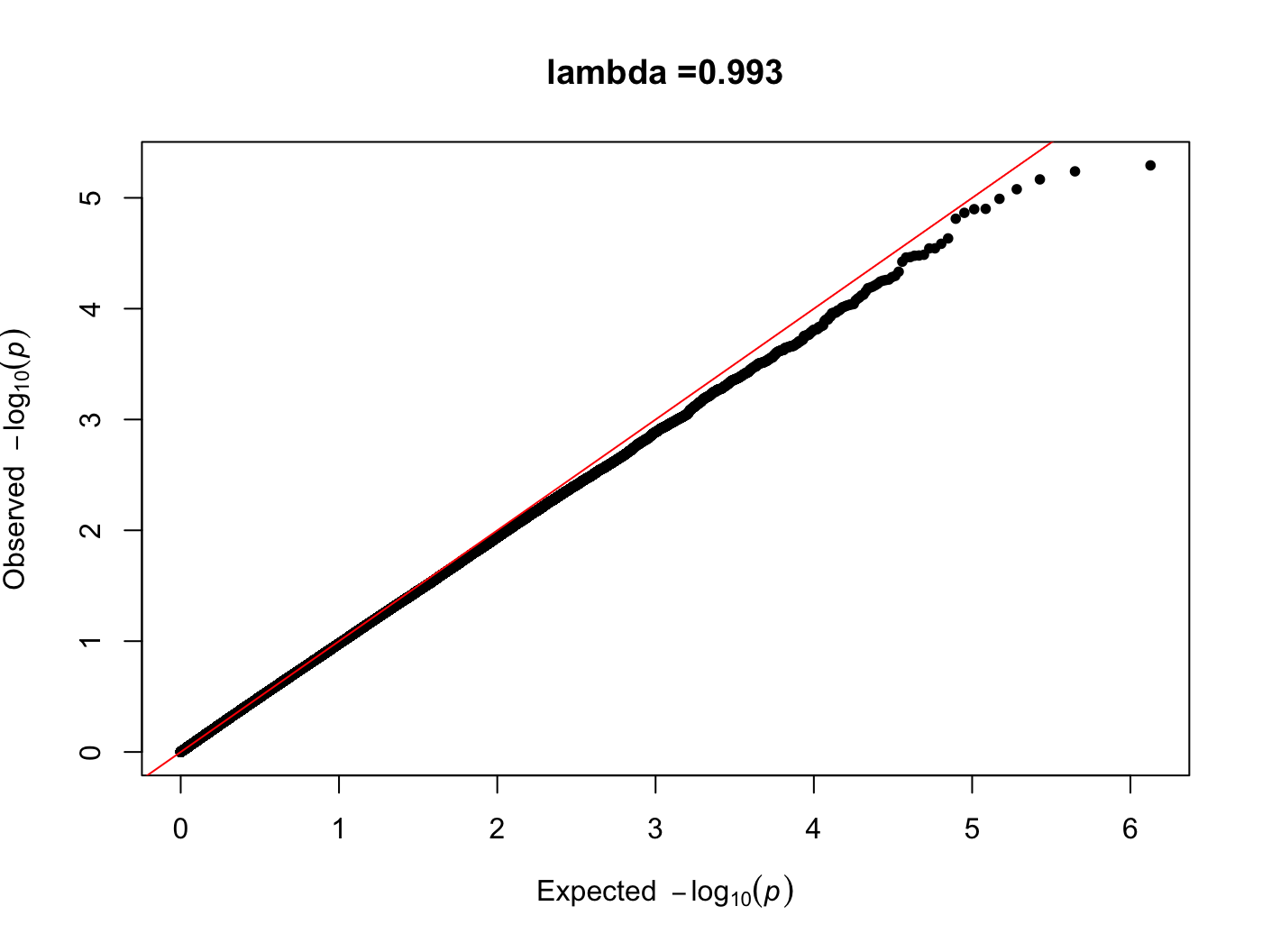


C): paired within-individual analysis for remission


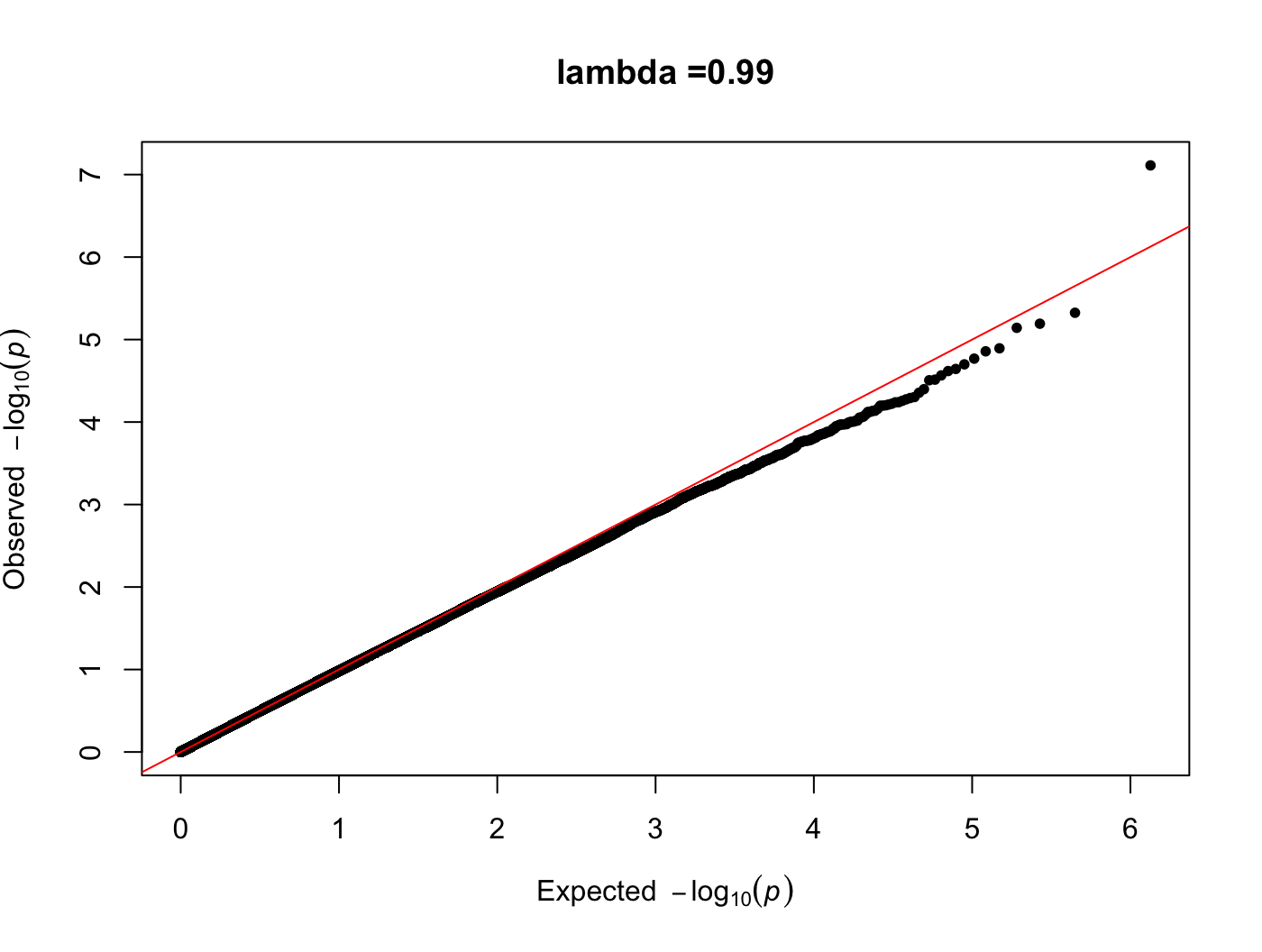


D): paired within-individual analysis for the stable course


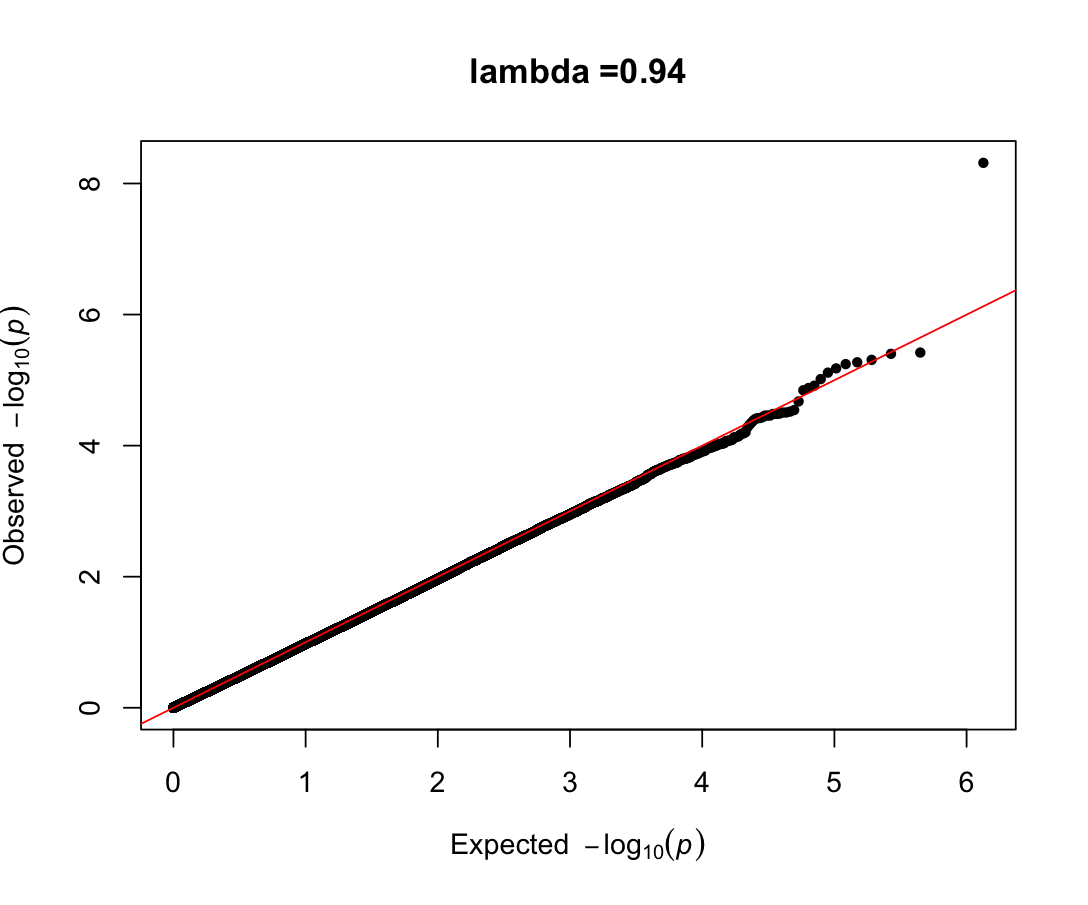


**Supplementary Figure S4: Correlogram response at 8 weeks**


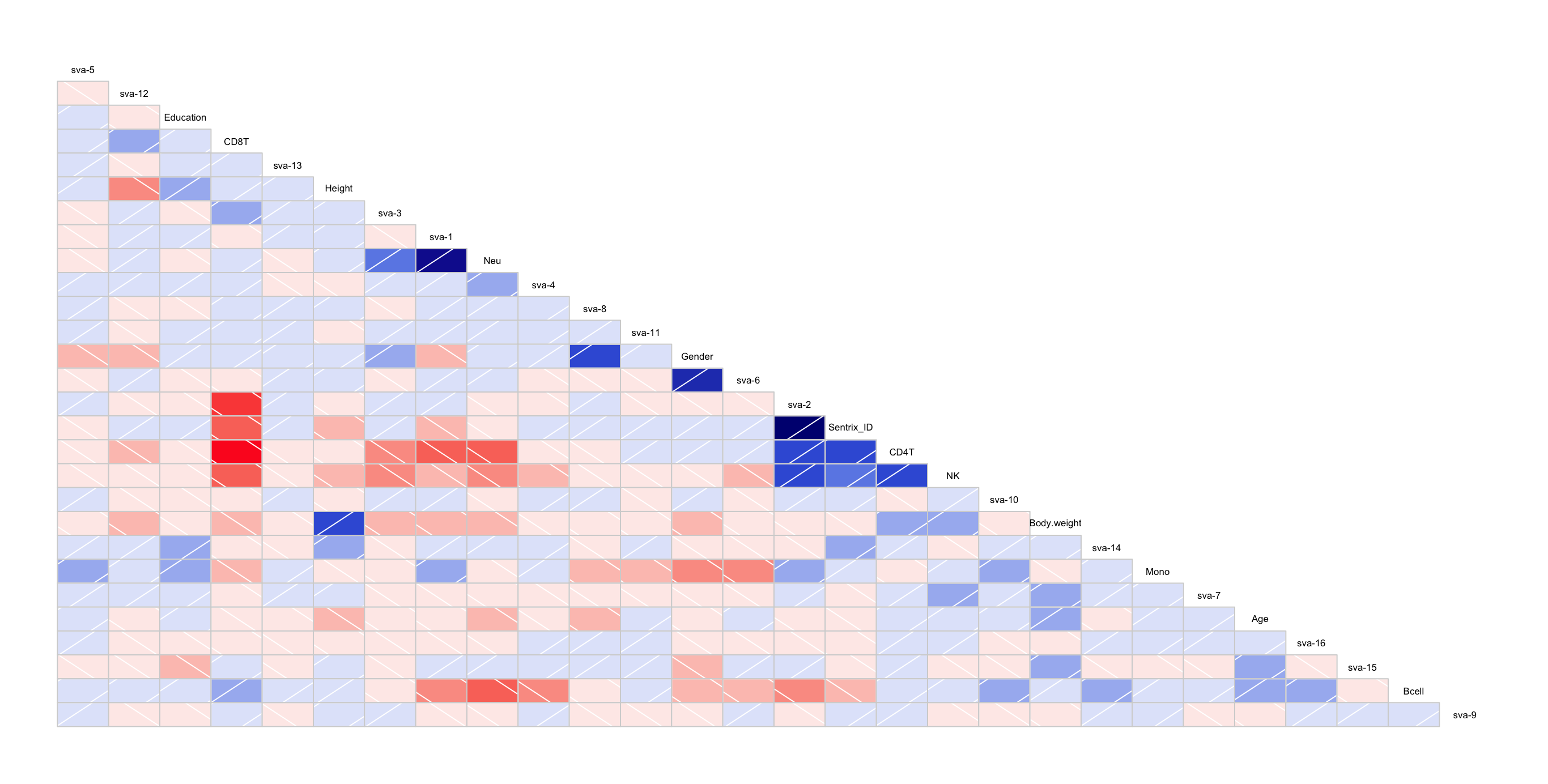


**Figure S5, Table S3, S4. Follow-up analysis responders-non responders at baseline**


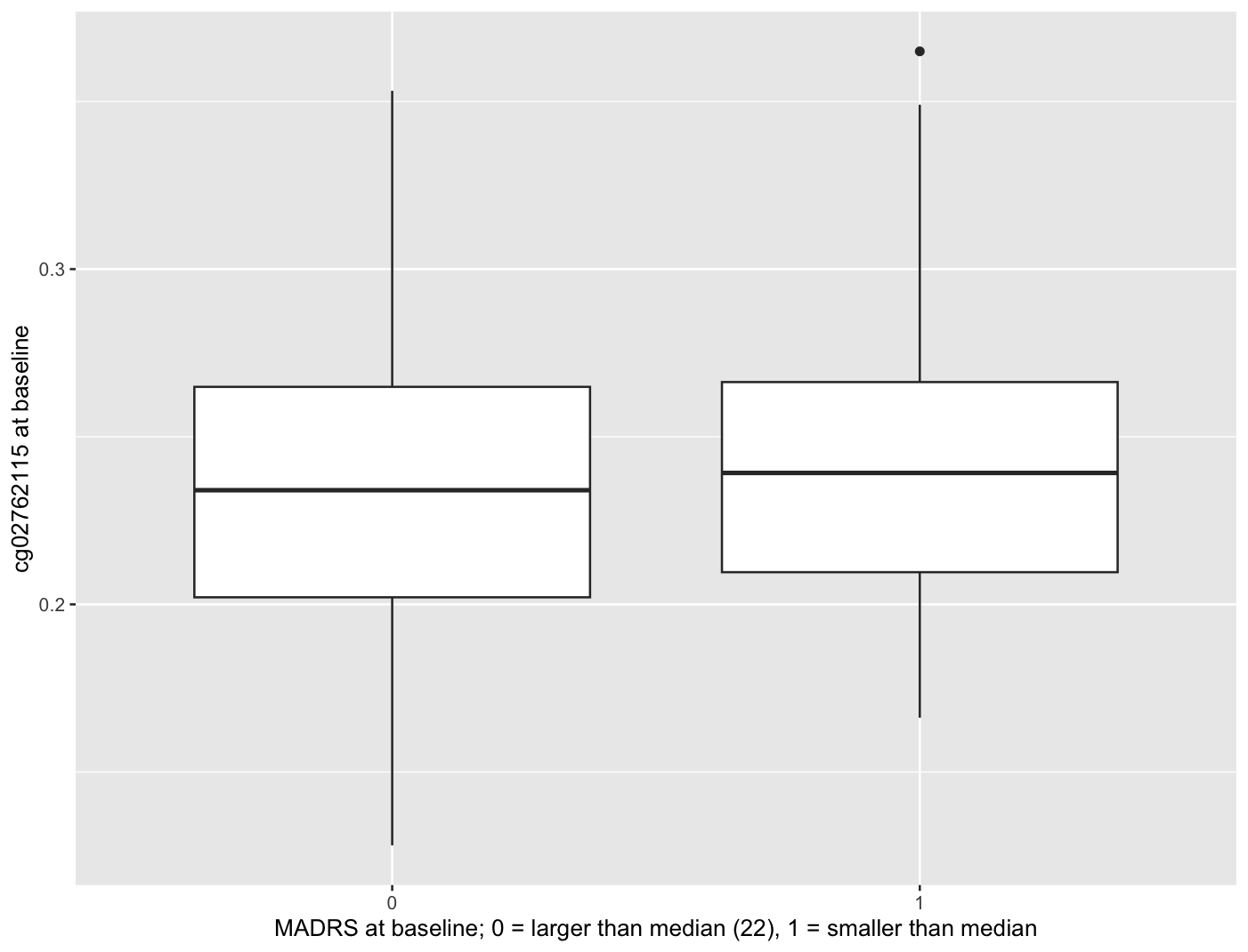

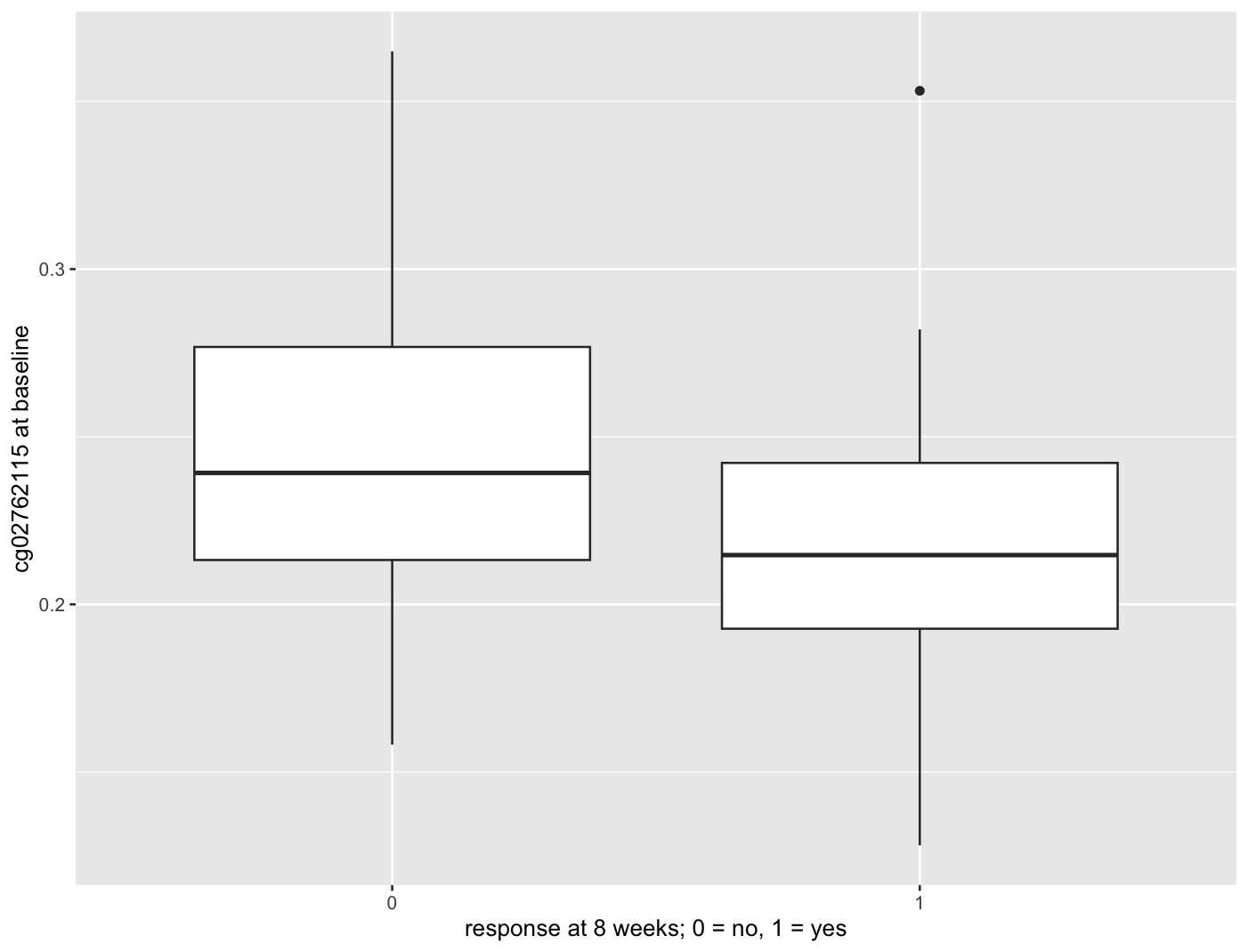


**Figure S5 DNA methylation at baseline for cg02762115: MADRS at baseline (divided by median; left), response at W8 (right)**

Model tested MADRS at baseline: lm(MADRS_BL ~ cg + Neu + NK + Bcell + CD4T + CD8T + Mono + Sex + Education + Body.weight + Height + Age)

|  | Estimate | Std. Error | t value | Pr(>\|t\|) |
| --- | --- | --- | --- | --- |
| cg26491461 | 29,92 | 75,15 | 0,40 | 0,69 |
| cg09148738 | 44,21 | 178,49 | 0,25 | 0,81 |
| cg01601845 | 43,84 | 36,21 | 1,21 | 0,23 |
| cg26340532 | -52,77 | 90,46 | -0,58 | 0,56 |
| cg25153882 | 44,40 | 65,69 | 0,68 | 0,50 |
| cg01388620 | -85,53 | 63,53 | -1,35 | 0,18 |
| cg05625299 | -32,43 | 48,63 | -0,67 | 0,51 |
| cg01002264 | 25,77 | 93,80 | 0,28 | 0,78 |
| cg02762115 | -20,49 | 21,71 | -0,94 | 0,35 |
| cg12083535 | -37,17 | 59,19 | -0,63 | 0,53 |

**Table S3: Results of baseline methylation and MADRS at baseline**

Model tested Response – non-response: aov(response ~ cg + Neu + NK + Bcell + CD4T + CD8T + Mono + Sex + Education + Body.weight + Height + Age + MADRS_BL)

|  | Df | Sum Sq | Mean Sq | F value | Pr(>F) |  |
| --- | --- | --- | --- | --- | --- | --- |
| cg26491461 | 1,00 | 0,17 | 0,17 | 0,89 | 0,35 |  |
| cg09148738 | 1,00 | 0,08 | 0,08 | 0,44 | 0,51 |  |
| cg01601845 | 1,00 | 0,02 | 0,02 | 0,10 | 0,76 |  |
| cg26340532 | 1,00 | 0,01 | 0,01 | 0,08 | 0,79 |  |
| cg25153882 | 1,00 | 0,06 | 0,06 | 0,32 | 0,58 |  |
| cg01388620 | 1,00 | 0,16 | 0,16 | 0,86 | 0,36 |  |
| cg05625299 | 1,00 | 0,53 | 0,53 | 2,92 | 0,092 | . |
| cg01002264 | 1,00 | 0,40 | 0,40 | 2,16 | 0,15 |  |
| cg02762115 | 1,00 | 0,89 | 0,89 | 4,88 | 0,030 | * |
| cg12083535 | 1,00 | 0,37 | 0,37 | 2,01 | 0,16 |  |

**Table S4: Results of baseline methylation and response after 8 weeks**

**Fig S6: overlap of CpGs within top 1% for all longitudinal analyses**

18

93

80

1403

**Supplementary Table S5: CpGs with *p* < 3x10^-5^: paired response analysis, top 13**

| CpGs | Chromosome | Start | CGI Relation | Mean diff (%) | Diffmeth. *p*-value | Gene |
| --- | --- | --- | --- | --- | --- | --- |
| cg22274825 | chr6 | 21593881 | North Shore | -1.94 | 5,10E-06 | SOX4 |
| cg06545910 | chr19 | 12846841 | North Shore | -1.96 | 5,77E-06 | C19orf43 |
| cg15982700 | chr7 | 27154911 | North Shore | 1.94 | 6,82E-06 | HOXA3 |
| cg14201528 | chr3 | 9791684 | Island | 0.98 | 8,36E-06 | OGG1 |
| cg04128358 | chr3 | 9167007 | Open Sea | -1.75 | 1,02E-05 | SRGAP3 |
| cg06825163 | chr1 | 202172912 | Open Sea | -3.70 | 1,26E-05 | LGR6 |
| cg17816193 | chr17 | 1946842 | South Shore | -1.70 | 1,27E-05 | DPH1 |
| cg25664220 | chr3 | 72788482 | South Shore | -2.09 | 1,37E-05 | Gene desert |
| cg19826686 | chr5 | 153694234 | Open Sea | 1.74 | 1,54E-05 | GALNT10 |
| cg27139956 | chr10 | 45470290 | Island | -1.06 | 2,32E-05 | RASSF4 |
| cg02330434 | chr8 | 111847388 | Open Sea | 2.74 | 2,60E-05 | Gene desert |
| cg10709267 | chr14 | 76841886 | North Shore | 1.54 | 2,86E-05 | ESRRB |
| cg23516589 | chr10 | 70718981 | South Shelf | 3.29 | 2,86E-05 | DDX21 |

*Negative values (Mean diff) = methylation increased over the course of 8 weeks; T0 < T1.

**Supplementary Table S6: CpGs with *p* < 3x10^-5^: paired remission analysis, top 11**

| CpGs | Chromosome | Start | CGI Relation | Mean diff (%) | Diffmeth. *p*-value | Gene |
| --- | --- | --- | --- | --- | --- | --- |
| cg02327902 | chr19 | 36239256 | North Shore | -1,23 | 7,74E-08 | LIN37 |
| cg18193817 | chr3 | 46606299 | North Shore | -1,52 | 4,73E-06 | LRRC2 |
| cg04061117 | chr3 | 9594385 | North Shore | 2,20 | 6,43E-06 | LHFPL4 |
| cg13359332 | chr1 | 46009622 | Open Sea | -0.58 | 7,21E-06 | Gene desert |
| cg03078057 | chr7 | 55415594 | South Shelf | 1,30 | 1,28E-05 | Gene desert |
| cg03062881 | chr21 | 47844012 | North Shore | -2,56 | 1,39E-05 | PCNT |
| cg12180984 | chr14 | 76819168 | Island | -1,02 | 1,70E-05 | Gene desert |
| cg13527872 | chr9 | 90114037 | South Shore | 2,54 | 2,00E-05 | DAPK1 |
| cg17793891 | chr17 | 43190922 | Open Sea | 1,44 | 2,27E-05 | PLCD3 |
| cg08854560 | chr22 | 38453474 | Island | -0.91 | 2,41E-05 | PICK1 |
| cg23516589 | chr10 | 70718981 | South Shelf | 3,20 | 2,72E-05 | DDX21 |

*Negative values (Mean diff) = methylation increased over the course of 8 weeks; T0 < T1.

**References**

1. Purcell S, Neale B, Todd-Brown K, et al. PLINK: A Tool Set for Whole-Genome Association and Population-Based Linkage Analyses. *The American Journal of Human Genetics*. 2007;81(3):559-575. doi:10.1086/519795

2. Anderson C a, Pettersson FH, Clarke GM, Cardon LR, Morris AP, Zondervan KT. Data quality control in genetic case-control association studies. *Nature protocols*. 2010;5(9):1564-1573. doi:10.1038/nprot.2010.116

3. Salas LA, Koestler DC, Butler RA, et al. An optimized library for reference-based deconvolution of whole-blood biospecimens assayed using the Illumina HumanMethylationEPIC BeadArray. *Genome Biol*. 2018;19(1):64. doi:10.1186/s13059-018-1448-7
